# Hydrogen Occupancy, Site Hierarchy, and Hydride-Transformation Pathways in BCC High-Entropy Alloys

**DOI:** 10.3390/molecules31101625

**Published:** 2026-05-12

**Authors:** Chen Chen, Quanhui Hou, Liangjuan Gao, Zhao Ding

**Affiliations:** 1 Department of Mechanics, Jinzhong University, Jinzhong 030606, China; chenchentgzy@163.com; 2 School of Automotive Engineering, Yancheng Institute of Technology, Yancheng 224051, China; hqhdyx66@ycit.edu.cn; 3 College of Materials Science and Engineering, Sichuan University, Chengdu 610065, China; lgao87@scu.edu.cn; 4 College of Materials Science and Engineering, National Engineering Research Center for Magnesium Alloys, National Innovation Centre for Industry-Education Integration of Energy Storage Technology, Chongqing University, Chongqing 400044, China

**Keywords:** high-entropy alloys, BCC structure, hydrogen occupancy, site hierarchy, hydride transformation, solid-state hydrogen storage

## Abstract

Body-centered cubic (BCC) high-entropy alloys (HEAs) are among the most promising HEA-based solid-state hydrogen-storage materials, yet their behavior is still too often discussed through composition, average phase label, or storage capacity alone. This Perspective argues that such descriptions remain incomplete because hydrogen accommodation in BCC HEAs is governed by the interplay among local interstitial accessibility, site hierarchy, and hydrogen-induced structural evolution. We therefore recast the problem around three linked questions: where hydrogen resides first, how the relative accessibility of tetrahedral and octahedral environments evolves with loading, and how that evolving occupancy redirects the host lattice toward specific hydride-transformation pathways. Recent experimental and computational studies show that hydrogen occupation in BCC HEAs is mixed, selective, and concentration-dependent, rather than fixed to a single ideal interstitial type. They also show that direct BCC-to-FCC/BCT-type hydrogenation routes, as well as pathway failure in structurally unstable BCC-related systems, are best understood from this occupancy-centered viewpoint. On this basis, we suggest that future design of BCC HEA hydrides should move beyond composition screening toward an occupancy-informed framework in which local site hierarchy, pathway integrity, and hydrogen-induced phase switching are treated as central design variables.

## 1. Introduction

**Figure 1:**
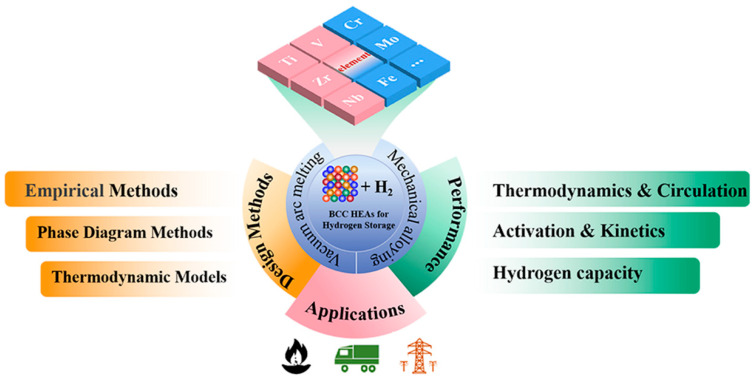
Overview of the design space, representative synthesis routes, hydrogen-storage properties, and broader application context of BCC-structured high-entropy alloys. Adapted from Ref. [4].

Solid-state hydrogen storage remains one of the most actively studied routes for hydrogen-energy deployment because it combines intrinsic safety with high volumetric storage density. Within this broader field, high-entropy alloys (HEAs) have attracted sustained attention as an emerging class of hydrogen-storage materials, since their multicomponent nature gives access to structural and thermodynamic states that are difficult to realize in conventional binary or ternary systems [1,2]. Among the different structural families explored so far, body-centered cubic (BCC) HEAs have become especially important. Recent reviews have already made it clear that BCC-structured HEAs form one of the most promising branches of HEA hydrogen-storage research because they repeatedly show favorable combinations of hydrogen capacity, activation behavior, kinetics, and structural tunability [3,4]. A broader overview of the design space, representative preparation routes, major hydrogen-storage responses, and the position of BCC HEAs within the wider HEA hydrogen-storage field is summarized in Figure 1, which serves here as the starting map for the subsequent discussion of hydrogen occupancy, site hierarchy, and hydride-transformation pathways.

Compared with conventional binary or ternary hydrides, BCC high-entropy alloys do not simply extend compositional complexity within an otherwise familiar host. Once severe local lattice distortion, chemically non-equivalent nearest-neighbor shells, and hydrogen-sensitive phase stability are introduced simultaneously, the same average BCC label may conceal very different hydrogen-hosting scenarios. Some alloys preserve a coherent route from initial site occupation to hydride formation and release, whereas others fragment into less reversible reaction networks despite broadly similar compositional motifs. This is why the present Perspective moves beyond asking which BCC HEAs absorb hydrogen and instead asks which of them maintain a structurally intelligible pathway linking local occupancy to reversible transformation. In this sense, Figure 1 serves not as a catalogue of alloy families, but as a map of how broad design space progressively narrows toward the specific structural questions that dominate hydrogen accommodation in BCC HEAs.

At the same time, current discussion of BCC HEAs still remains weighted toward composition and macroscopic performance. Much of the literature is organized around which alloy family stores more hydrogen, which alloy activates more easily, or which alloy gives a more attractive pressure–composition plateau. These questions are important, but they do not yet reach the structural level at which hydrogen storage in BCC HEAs is actually decided. Hydrogen does not interact with an alloy through nominal composition alone, but nominal composition remains the necessary starting point for design. A practical entry into BCC high-entropy-alloy discovery is still composition-first: one begins with alloy families that satisfy established BCC-forming tendencies and hydrogen-relevant constraints, including appropriate valence electron concentration and sufficient, but not destructive, atomic-size mismatch [3,4,5,6]. What nominal composition cannot do by itself is predict how hydrogen will rank the many local interstitial environments that emerge once chemical disorder and lattice distortion are introduced.

In this Perspective, the term interstitial landscape is used to describe that distributed set of hydrogen-accessible local environments, including the geometry of the available interstices, the nearest-neighbor chemical shell, the local strain state, and the relative energetic ranking among competing sites. The word landscape is intentional: hydrogen in a BCC high-entropy alloy does not encounter one ideal tetrahedral or octahedral site repeated periodically, but a spectrum of locally nonequivalent sites whose accessibility and stability evolve with pressure, temperature, and hydrogen content [7,8,9,10]. This is why two alloys with the same average structure may still display markedly different occupation sequences, plateau behavior, and hydride-transformation routes. This point is not merely conceptual. Experimental work on TiVZrNbHf has shown that hydrogen uptake in a BCC HEA can exceed the range normally associated with ordinary transition-metal hydrides and is accompanied by a distinct hydrogenation route that differs from the conventional picture for simpler BCC hosts. The same study explicitly related the unusually high H/M ratio to a strained lattice environment that could favor hydrogen accommodation in both tetrahedral and octahedral sites [5]. Later analyses summarized in the literature further indicate that hydrogen occupancy in BCC HEAs is not restricted to a single ideal interstitial type. Instead, tetrahedral and octahedral occupation may coexist, with their relative importance depending on thermodynamic condition and hydrogen loading. The implication is direct: the BCC host in a high-entropy alloy is not a uniform lattice with one predetermined hydrogen site preference, but a structurally and chemically heterogeneous network in which site accessibility itself becomes part of the storage problem [3,5].

Computational studies strengthen this interpretation. In TiZrHfScMo, first-principles calculations show that hydrogenation is energetically favorable over a substantial composition range, but also that formation enthalpy, binding energy, and metal–hydrogen bonding evolve systematically with increasing hydrogen content. Different constituent elements do not contribute equally to hydrogen uptake, and the character of the hydrogen-host interaction changes as loading proceeds [6]. This means that hydrogen occupancy in BCC HEAs should not be treated as a static structural label. It is better understood as a concentration-dependent process in which local bonding, lattice expansion, and interstitial accessibility co-evolve. Once that point is recognized, the transition from hydrogen occupancy to hydride-transformation pathway becomes the natural center of the discussion rather than a secondary detail.

A further reason to adopt this perspective is that not every nominally BCC-related alloy provides a viable hydrogen-hosting route. Mechanically synthesized Mg-containing systems already show that a design that appears favorable at the level of composition may still fail to sustain a coherent hydrogen-storage pathway if low crystallinity, amorphization, or structural instability intervene. In MgVCr, a BCC solid-solution phase can still be identified and the alloy retains reasonable structural stability during cycling, whereas MgTiVCrFe shows coexistence of crystalline and amorphous regions together with poor hydrogen-storage performance and partial decomposition after cycling [7]. In other words, the BCC label is necessary to define an important class of hosts, but it is not sufficient to explain performance. The decisive issue is whether the alloy supports a coherent sequence linking initial site occupancy, hydrogen-induced structural response, and reversible release. This is why a pathway-centered approach is needed: it sharpens the distinction between alloys that merely contain hydrogen-accessible structural motifs and alloys that can sustain an effective hydrogen-hosting route [7].

The purpose of this perspective is therefore not to provide another general review of HEAs for hydrogen storage. Instead, it focuses on a narrower and more structural question: how should hydrogen storage in BCC HEAs be interpreted when attention is centered on interstitial occupation and hydrogenation route? To address this question, the discussion first considers why the BCC framework is uniquely relevant to hydrogen accommodation in multicomponent alloys, then evaluates the evidence for mixed and concentration-dependent occupancy, and finally considers how occupancy-controlled transformation pathways define both successful and unsuccessful hydrogen-storage behavior in representative systems. By framing the problem in this way, the article aims to shift the discussion of BCC HEA hydrogen storage from composition screening toward a more mechanistic, occupancy-informed design logic.

## 2. Structural Basis of Hydrogen Accommodation in BCC High-Entropy Alloys

The special importance of body-centered cubic high-entropy alloys in hydrogen storage does not arise simply from the fact that they belong to the broader HEA family. It arises from the kind of hydrogen-hosting framework that the BCC lattice provides once multicomponent alloying, lattice distortion, and local chemical heterogeneity are introduced together. Recent reviews have already made it clear that BCC HEAs occupy a particularly important position within HEA-based hydrogen storage because they repeatedly combine relatively high hydrogen capacity with structurally diverse hydrogenation behavior. Yet the real significance of the BCC framework is not exhausted by capacity alone. What makes it distinctive is that it presents hydrogen with a comparatively open interstitial network whose accessibility is no longer fixed once multiple principal elements, local strain, and uneven chemical environments are all present in the same host lattice [3,4]. The hydrogen-storage problem in BCC HEAs is therefore not simply whether hydrogen can enter the alloy, but what kind of site hierarchy the host actually presents once this structural complexity is taken into account.

In a simplified crystallographic description, hydrogen in BCC-related systems is often discussed in terms of tetrahedral and octahedral interstitial sites. For BCC HEAs, however, these sites should not be treated as rigidly equivalent or uniformly accessible throughout the lattice. One reason is geometric. Atomic-size mismatch among constituent elements produces local distortion, so the effective interstitial volumes are distributed rather than identical. Another reason is chemical. The local environment around a nominally similar site may vary substantially depending on which elements coordinate it. This means that hydrogen in a BCC HEA does not encounter one ideal BCC matrix, but a spectrum of locally distinct environments. The consequence is fundamental: site preference in these alloys is not a simple crystallographic fact but a structurally conditioned hierarchy. Some sites become more accessible, some are destabilized, and some may only become favorable once hydrogen loading or lattice expansion reaches a certain level [5,8,9,10].

**Figure 2:**
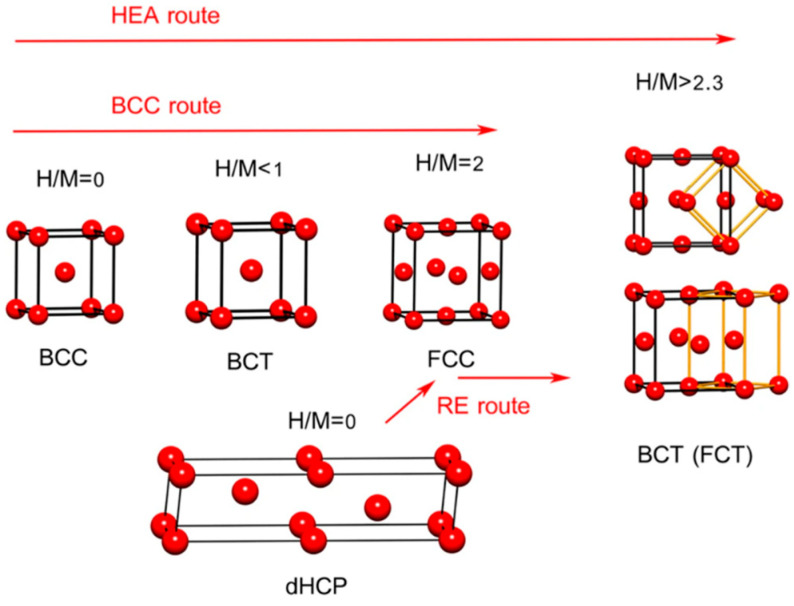
Schematic comparison of hydrogen-hosting routes in metallic systems, including the conventional BCC route, the rare-earth route, and the route observed in a representative high-entropy alloy. The figure emphasizes the distributed local environments that define the interstitial landscape in BCC HEAs. Adapted from Ref. [7].

This is why lattice strain must be treated as more than a generic descriptor of disorder. In conventional discussion, strain is often invoked as a broad explanation for why HEAs may behave differently from simpler alloys. In the present context, its significance is more specific. Strain changes the geometry of the interstitial landscape and therefore changes which hydrogen-hosting route is even available to the alloy. The TiVZrNbHf system provided one of the clearest early indications of this point: its unusually high hydrogen uptake was linked to a highly strained host lattice that could favor hydrogen accommodation in both tetrahedral and octahedral environments [5]. More specifically, the unusually high hydrogen uptake in TiVZrNbHf has been associated with a highly strained BCC host that broadens the distribution of accessible interstitial environments, thereby allowing mixed tetrahedral-octahedral participation under appropriate thermodynamic conditions rather than confinement to a single ideal site family. Subsequent analyses have also shown that local descriptors such as valence electron concentration cannot be understood only at the level of average composition. At the local scale, the fraction of occupied interstitial sites depends on the VEC of the nearest-neighbor environment, indicating that site stability in BCC HEAs is tied not only to void size but also to the electronic character of the surrounding atoms [8,9]. In this sense, the BCC framework in HEAs is special not because it offers “more space” in a simple geometric sense, but because it creates a deformable and chemically nonuniform interstitial network whose site hierarchy can be redistributed by both strain and local bonding. The conceptual contrast between a conventional BCC hydride picture and the interstitial-landscape perspective adopted here is summarized schematically in Figure 2, highlighting that hydrogen accommodation in BCC HEAs should be interpreted through distributed local environments rather than a single idealized site model.

A further consequence of this picture is that BCC HEAs should not be regarded as merely enlarged versions of conventional BCC hydrides. In simpler BCC alloy systems, hydrogen accommodation can often be described with a more uniform site model and a more predictable sequence of hydride formation. In BCC HEAs, by contrast, the interplay among interstitial topology, lattice strain, and local chemical heterogeneity makes the occupancy landscape inherently distributed. This does not make the BCC label unimportant; rather, it changes what the label means. Here, BCC no longer signifies only a broad structural category associated with favorable hydrogen accommodation. It signifies a host framework in which the hierarchy of accessible sites, the energetic order in which those sites are filled, and the tendency of the lattice to switch into a new hydrogenated structure are all modulated by multicomponent local structure [3,4,9,10]. The practical implication is that hydrogen storage in BCC HEAs cannot be reduced to the question of whether a BCC phase is present. What matters is what kind of BCC-based hydrogen-hosting landscape the alloy actually realizes.

This is why a pathway perspective becomes necessary at a very early stage of the discussion. Once the BCC host is recognized as a variable interstitial landscape rather than a fixed structural container, hydrogen occupancy can no longer be treated as a simple site label assigned once and for all. Instead, it becomes a conditional structural process that depends on local site hierarchy, hydrogen concentration, and the evolving response of the host lattice itself. The next section therefore turns from the BCC framework in general to a more direct question: what evidence is there that hydrogen occupancy in BCC HEAs is mixed, selective, and concentration-dependent rather than unique and fixed? Representative systems discussed below, together with their reported local-environment features, site-occupation characteristics, transformation behavior, and hydrogen-storage response, are summarized in Table 1.

## 3. Hydrogen Occupancy in BCC High-Entropy Alloys: Mixed Sites, Local Selection, and Concentration-Dependent Evolution

If the previous section establishes why the BCC framework is structurally special, the next question is whether hydrogen occupancy in these alloys can in fact be treated as a variable rather than as a fixed crystallographic attribute. This is the point at which the conventional simplification begins to break down. In many hydrogen-storage discussions, especially for simpler alloy systems, it is tempting to speak of one preferred interstitial type as though hydrogen simply fills a predetermined site family until the host approaches saturation. For BCC high-entropy alloys, however, the available evidence points to a more complex situation. Hydrogen occupancy is not adequately described by a single idealized site preference. Instead, it depends on local site geometry, nearest-neighbor chemistry, hydrogen concentration, and the structural state into which the alloy is evolving during absorption [3,5,6,8,9,10]. What must therefore be examined is not only whether tetrahedral or octahedral sites are occupied, but under what conditions, to what extent, and with what consequences for subsequent structural evolution.

### 3.1. Mixed Tetrahedral–Octahedral Occupancy

The clearest evidence that hydrogen occupancy in BCC HEAs is not unique comes from the TiVZrNbHf family. Early work on TiVZrNbHf drew attention because the alloy exhibited an unusually high hydrogen-to-metal ratio together with a hydrogenated structure that could not be explained by a one-site picture alone [5]. Subsequent neutron-diffraction studies on deuterated TiVZrNbHf showed more directly that deuterium can occupy both tetrahedral and octahedral interstitial sites. Under high-temperature and high-pressure conditions, the reported occupation was approximately balanced between the two site families, whereas ex situ measurements at room temperature showed a much stronger preference for tetrahedral occupation [13,14]. These observations are important because they demonstrate that mixed occupancy is not a speculative correction to the BCC model. It is an experimentally observable feature of hydrogenated BCC HEAs, and its apparent balance shifts with thermodynamic condition rather than remaining fixed.

This point also changes how hydrogen-storage behavior should be interpreted. In a simpler host, one may reasonably ask which site is preferred. In a BCC HEA, that question is still valid, but it is no longer sufficient. The more useful question is how the occupation of available sites redistributes as the alloy moves from one structural and thermodynamic state to another. Site preference is therefore conditional rather than absolute. Even when tetrahedral occupation becomes dominant under one condition, the system may still retain a measurable octahedral contribution or move toward a different site balance as hydrogen content, temperature, or pressure change [3,13,14]. This is why hydrogen occupancy in these alloys should be treated as a state-dependent structural variable rather than a static site label.

At the same time, mixed tetrahedral–octahedral occupancy should not be presented as a phenomenon exclusive to high-entropy alloys. Related behavior has also been discussed in more conventional alloy systems. For example, recent first-principles analysis of a metastable BCC TiVCr medium-entropy alloy showed that hydrogen can be accommodated in both octahedral and tetrahedral sites, with the preferred occupation pattern depending on structural state and local bonding conditions [16]. This comparison is important because it places BCC HEAs in a broader metallic-hydride context. What is distinctive in BCC HEAs is therefore not simply the existence of both site families, but the way multicomponent chemical disorder, local lattice distortion, and loading-dependent structural evolution redistribute their relative accessibility and stability.

### 3.2. Local Site Inequivalence and Effective Site Availability

A second important lesson from the same body of work is that not all nominally similar sites in a BCC HEA are equally accessible. Reverse Monte Carlo analysis of hydrogenated TiVZrNbHf showed that deuterium tends to avoid the smaller tetrahedral volumes, consistent with the Westlake criterion that an interstitial site must exceed a minimum effective volume in order to stably host hydrogen [14]. Because atomic-size mismatch in HEAs makes some local environments more compressed than others, the tetrahedral population is intrinsically distributed rather than uniform. Under these conditions, partial occupation of octahedral sites is no longer anomalous in a simple geometric sense; it is a natural consequence of local site inequivalence. In other words, what matters is not only whether a site is classified as tetrahedral or octahedral by average crystallography, but whether a given local realization of that site remains large enough and chemically favorable enough to host hydrogen.

Accordingly, the occupancy problem in BCC HEAs is better described as competition among a spectrum of locally realized interstitial environments than as a simple competition between two ideal site classes. Once local distortion and local chemistry are taken into account, the effective site hierarchy becomes distributed rather than uniform, which helps explain why alloys sharing the same average BCC label can still display different hydrogen-storage responses and reversibility.

### 3.3. Local Electronic Environment and Site Stability

A third layer of complexity comes from the electronic environment around each interstitial. Work on BCC HEAs has shown that site stability is influenced not only by geometric size but also by the valence electron concentration of the nearest-neighbor metal atoms. Modeling of TiVNbD5.7 indicated that the fraction of occupied interstitials with a given local coordination environment decreases as the average VEC of the nearest neighbors increases. In other words, interstitials surrounded by lower-VEC environments are more likely to remain occupied, whereas sites coordinated by higher-VEC metal atoms are preferentially depopulated first during hydrogen release [8,13]. This is a particularly important result because it shows that local electronic structure and local occupancy are directly linked. A site may appear geometrically available, yet still differ in stability depending on the chemical identity of the four or more metal atoms that define it. This local VEC dependence is consistent with the broader TiVZrNbHf-based literature, in which variations in local lattice strain and nearest-neighbor chemistry measurably shift hydrogen sorption behavior even when the average BCC phase label is retained.

This perspective helps move the field beyond broad statements about multicomponent synergy. If site occupation depends measurably on local VEC, then occupancy in BCC HEAs is not only a structural consequence of disorder; it is a chemically resolved property of the local environment. This is one reason why average descriptors such as global VEC, atomic-size mismatch, or average phase label cannot on their own provide a complete explanation of hydrogen-storage behavior. They remain useful as broad design guides, but the actual occupancy process is governed at a finer scale. For the purposes of the present perspective, this means that site hierarchy in BCC HEAs should be viewed as a local electronic–geometric hierarchy rather than as a purely crystallographic one.

### 3.4. Concentration-Dependent Occupancy Evolution

Hydrogen occupancy in BCC HEAs is also concentration-dependent. This point is especially clear in theoretical work on TiZrHfMoNb, which indicates that the octahedral sites of the BCC phase are preferentially occupied at low hydrogen concentration, whereas at hydrogen contents above about 1.08 wt.% H2 the host structure transforms to FCC and the tetrahedral sites of the FCC phase become preferentially occupied [15]. This result is conceptually important for two reasons. First, it shows that site preference can shift as hydrogen loading proceeds, even within the same alloy. Second, it shows that the occupancy problem cannot be separated from the phase-transformation problem. Occupancy is not a question to be answered first and then set aside; it is the mechanism through which the host lattice is progressively pushed into a new hydrogenated structural state.

Computational studies on TiZrHfScMo point to the same conclusion from a different direction. As hydrogen content increases, both the formation enthalpy and the binding-energy landscape evolve systematically, and the relative contributions of different metal–hydrogen interactions also change [6]. This means that the host lattice is not responding to hydrogen in a fixed way over the entire loading range. Rather, each increment of hydrogen changes the conditions for the next increment by altering local bonding, lattice expansion, and the relative favorability of subsequent site occupation. Under these conditions, hydrogen occupancy is better understood as a concentration-dependent structural process than as a one-time site assignment. In this alloy, the significance is therefore not merely that hydrogen absorption is energetically favorable, but that the driving force for further uptake, the preferred local bonding partners, and the effective site hierarchy are all reconfigured as loading proceeds.

### 3.5. Occupancy as a Pathway Variable

Taken together, the available evidence suggests that hydrogen occupancy in BCC HEAs should not be reduced to a one-site model. Mixed tetrahedral–octahedral occupation, local exclusion of undersized tetrahedral volumes, local VEC effects, and concentration-dependent changes in preferred site family all indicate that occupancy is conditional, selective, and evolving [8,13,14,15]. This has a direct implication for how BCC HEA hydrides should be discussed. The relevant structural question is not simply which sites are occupied, but how the hierarchy of accessible sites changes as hydrogen loading proceeds and how that evolving hierarchy redirects the host lattice toward a specific hydrogenated state. That is why occupancy in these materials is best treated as a pathway variable rather than as a fixed crystallographic descriptor. Once this is recognized, the transition to the next section becomes natural: if site occupation evolves with hydrogen concentration, then hydride-transformation pathways are not separate from occupancy, but are its structural continuation.

## 4. Hydride-Transformation Pathways in BCC High-Entropy Alloys: Experiment, Energetics, and Structural Switching

If hydrogen occupancy in BCC high-entropy alloys is not unique, then the next question is how that evolving occupancy redirects the host lattice into specific hydrogenated structures. This is the point at which hydrogen storage ceases to be a matter of site filling alone and becomes a problem of pathway. In simpler metal–hydrogen systems, it is often possible to describe hydrogenation as a sequence linking a parent host to one or more hydride phases. In BCC HEAs, however, the situation is more structurally coupled. Site selection, lattice distortion, and hydrogen-induced phase switching are not independent events. They belong to the same evolving process, and the hydrogenated phase that appears at the end of absorption is best understood as the structural consequence of how the alloy has accommodated hydrogen along the way [3,6,13,14,15].

### 4.1. One-Step Hydrogenation and BCC-to-FCC Switching

A particularly instructive example is provided by the non-equimolar Al_0.1_Ti_0.3_V_0.25_Zr_0.1_Nb_0.25_ alloy. Under hydrogenation, this alloy shows a single equilibrium pressure plateau below 0.1 MPa at 298 K and transforms from a BCC host to an FCC hydride phase, with a gravimetric capacity of about 2.2 wt.% H_2_ together with improved cycling response compared with more conventional BCC hydrides [3,11]. This behavior is important because it contrasts with the classical V–H route, where hydrogenation proceeds through two stages and two plateau regions associated with successive BCC-derived hydrogenated states. In the present context, the significance of the Al-containing HEA is not simply that it stores hydrogen efficiently at room temperature. Its importance is that it shows, in an experimentally resolved way, that a multicomponent BCC host can follow a one-step BCC-to-FCC-type hydrogenation pathway that bypasses the less desirable two-stage route typical of simpler BCC alloys. At the level of structural shorthand, the reported reversible route may be written as BCC host + xH ↔ FCC hydride under the experimental conditions of Ref. [11].

**Figure 3:**
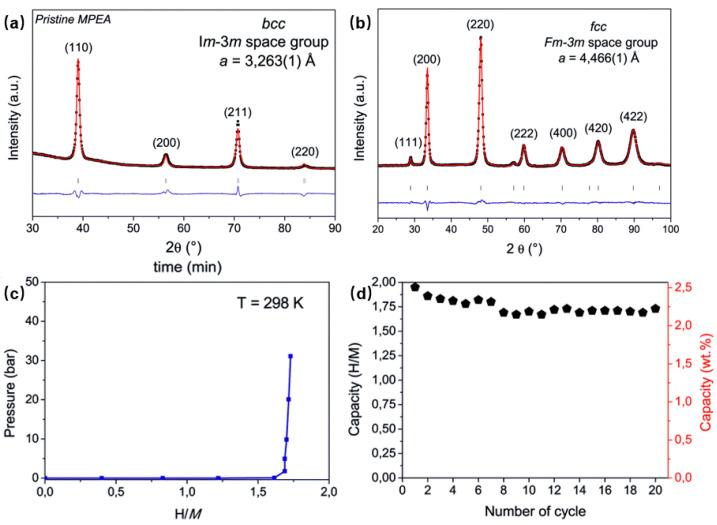
Experimentally resolved one-step hydrogenation pathway in a non-equimolar BCC high-entropy alloy: (**a**) XRD pattern of the as-cast BCC host alloy; (**b**) XRD pattern after hydrogenation, showing formation of an FCC hydride phase; (**c**) pressure-composition isotherm at 298 K, indicating a single equilibrium plateau; and (**d**) hydrogen-storage capacity upon absorption/desorption cycling. Adapted from Ref. [3].

This result changes how hydrogenation pathway should be discussed in BCC HEAs, but it should be interpreted with appropriate caution. The experimentally resolved feature is that Al_0.1_Ti_0.3_V_0.25_Zr_0.1_Nb_0.25_ follows a direct BCC-to-FCC hydrogenation route and exhibits a single clearly resolved plateau within the accessible pressure range. However, this should not be overgeneralized as proof that all apparently one-step BCC HEA transformations are fully free of unresolved intermediate behavior at lower hydrogen contents. In multicomponent hydrides, the equilibrium pressure in the low-H/M regime may in some cases fall below instrumental detection limits, so the apparent absence of an additional plateau does not necessarily exclude subtle intermediate occupancy or transformation processes. The more defensible conclusion is therefore narrower but stronger: relative to classical BCC hydrides, some BCC HEAs can follow a much more direct hydrogenation route into the final hydride state, even if the detailed low-loading sequence still requires case-by-case experimental resolution. This experimentally resolved direct hydrogenation route, together with its associated XRD, PCI, and cycling features, is shown in Figure 3, supporting the argument that some BCC HEAs can access a comparatively direct one-step hydrogenation pathway [3,11]. Taken together, these observations define a coupled pathway signature rather than a set of isolated measurements. The parent BCC diffraction state, the emergence of the FCC hydride, the single accessible plateau, and the retained cycling response all point to a hydrogenation route that remains structurally concentrated rather than being clearly partitioned into multiple resolved intermediate regimes. For the present Perspective, this case is important because it shows that a favorable BCC HEA is defined not only by how much hydrogen it stores, but by whether its occupancy sequence can be funneled into a direct and recoverable transformation route under experimentally relevant conditions.

### 4.2. Concentration-Driven Phase Switching

The one-step route, however, should not be interpreted as evidence that the occupancy problem disappears once the alloy begins to hydrogenate. On the contrary, it shows that occupancy and transformation are coupled throughout the process. This point becomes particularly clear when considering theoretical studies of TiZrHfScMo and TiZrHfMoNb, in which hydrogen content was treated as an explicit variable rather than as a before-and-after condition. In these systems, both formation enthalpy and binding energy evolve systematically with increasing hydrogen content, indicating that the energetic conditions for further uptake are modified by the hydrogen that is already present [6,15]. At lower loading, occupation of the available BCC-based interstitial landscape remains favorable. At higher loading, however, the host enters a new structural regime in which the original BCC framework is no longer the only relevant hydrogen-hosting state.

This concentration dependence is not merely thermodynamic. It also has a structural expression. Calculations on TiZrHfMoNb indicate that octahedral sites in the BCC phase are preferentially occupied at low hydrogen concentration, whereas once the hydrogen content exceeds about 1.08 wt.% H_2_ the structure converts to FCC, from which point the tetrahedral sites of the FCC phase become preferentially occupied [15]. This is a crucial result for the argument of the present article. It shows that occupancy and phase switching are not two separate topics. The preferred site family itself changes once the host lattice enters a different hydrogenated state. In other words, the pathway problem is an occupancy problem continued by structural transformation.

**Figure 4:**
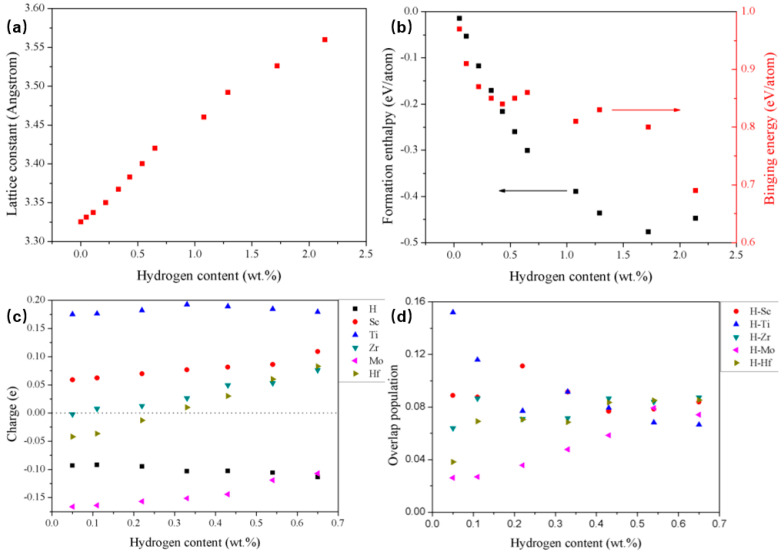
Energetic and electronic evolution of hydrogenated BCC high-entropy alloys with increasing hydrogen content, including changes in (**a**) lattice expansion, (**b**) formation enthalpy, (**c**) binding energy, and (**d**) metal-hydrogen bonding. Adapted from Ref. [6].

The same conclusion is supported from another angle by the energetic and electronic evolution observed in TiZrHfScMo. As hydrogen concentration increases, the lattice expands, formation enthalpy evolves non-monotonically, and the relative contribution of different metal–hydrogen bonds changes. Ti-associated bonding becomes relatively less favorable, while Hf- and Mo-associated interactions become more significant at higher hydrogen content [6]. These changes indicate that the host is not accommodating hydrogen in a structurally passive way. Each increment of hydrogen changes the conditions for the next increment by reshaping both the interstitial landscape and the local bonding hierarchy. Phase switching is therefore not an abrupt event appended after site filling; it is the cumulative structural response to concentration-dependent occupancy. The corresponding energetic and electronic evolution with increasing hydrogen content is summarized in Figure 4, showing that hydrogen uptake progressively reshapes both the bonding hierarchy and the structural stability of the host lattice. The implication is that phase stability in hydrogenated BCC HEAs cannot be inferred from the unloaded host lattice alone. As hydrogen concentration rises, the alloy continuously reorganizes its local energetic landscape, so the preferred hydride product emerges from progressive reweighting of metal–hydrogen interactions rather than from a fixed structural predisposition. In that sense, concentration-dependent energetics is part of pathway design itself, because it determines whether increasing hydrogen content reinforces or destabilizes the structural logic of the transformation route.

### 4.3. Pathway Integrity Versus Endpoint Phase Labels

Once these results are considered together, a more general conclusion follows. The representative cases behind this comparison are also organized in Table 1. In BCC HEAs, the hydrogenated phase should not be treated as the sole structural target of interpretation. Knowing that an alloy ends in an FCC- or BCT-related hydride state is useful, but not sufficient. What matters more fundamentally is how the alloy reaches that state: whether hydrogen first occupies mixed tetrahedral–octahedral environments, whether the BCC host sustains a coherent occupancy hierarchy as loading increases, and whether transformation proceeds through a direct and reversible route or through a less stable, more conventional multi-step sequence [3,5,6,11,15]. For this reason, the most informative descriptor is not the endpoint phase label alone, but the integrity of the hydrogenation pathway that links the BCC host to its hydrogenated product. Here, pathway integrity refers to the ability of an alloy to preserve a structurally intelligible and cyclically recoverable sequence from initial hydrogen occupation, through hydrogen-induced lattice response, to final hydride formation and subsequent dehydrogenation. A pathway has high integrity when this sequence remains coherent under repeated uptake and release, without being undermined by uncontrolled amorphization, severe phase fragmentation, or irreversible decomposition. In this sense, pathway integrity is not simply another name for reversibility. Rather, it denotes the structural condition that makes meaningful reversibility possible. This perspective also clarifies why alloys with broadly similar starting structures may still exhibit very different storage behavior. Two BCC hosts may differ not because one is “more BCC” than the other, but because one supports a direct route into a useful high-hydrogen state while the other is trapped in a less favorable sequence of intermediate occupancies and transformations. This is why contrast cases are essential: they define where a nominally BCC host no longer provides a viable hydrogen-hosting route.

## 5. Pathway Failure and Design Boundaries in BCC-Related Systems

The preceding sections have argued that hydrogen storage in BCC high-entropy alloys is best understood through the way hydrogen is accommodated locally and through the transformation route that connects the parent host to its hydrogenated product. That argument becomes more convincing when one also considers systems in which the pathway is weakened, interrupted, or never fully established. Such cases are important because they prevent the BCC label from being treated as a sufficient explanation. A compositionally complex alloy may still be described as BCC-related in design intent and yet fail to provide an effective or reversible hydrogen-storage route if the structurally realized state does not support coherent hydrogen occupancy or stable transformation. In this sense, lower-performing systems are not peripheral to the present perspective. They are necessary tests of whether the occupancy–pathway framework has real explanatory value [7,12,17,18,19].

### 5.1. MgVCr: A Limited but Coherent BCC-Derived Pathway

The MgVCr system provides a useful starting point because it shows that a mechanically synthesized alloy can retain a hydrogen-storage pathway that remains structurally legible even if the absolute reversible capacity is not especially high. After high-energy ball milling, MgVCr exhibits a BCC solid-solution phase; after hydrogenation and dehydrogenation cycling, the alloy still shows mainly the BCC phase together with a limited amount of MgH_2_, indicating that the BCC-derived host remains reasonably stable during cycling. Structurally, the key point is that the milled MgVCr powder retains a better-resolved BCC-derived diffraction signature during hydrogenation/dehydrogenation, indicating that hydrogen enters a host that remains crystallographically legible. At 350 °C, the alloy reaches about 0.95 wt.% hydrogen and displays a relatively clear pressure plateau, followed by complete desorption under the reported conditions [7]. These values are modest compared with the most hydrogen-rich refractory HEAs, but that is not the main point here. The significance of MgVCr is that it still preserves a recognizable route linking a BCC-related host, hydrogen uptake, and reversible release. In other words, the hydrogen-hosting pathway remains structurally coherent even though the storage window is limited.

This distinction matters because it shows that pathway integrity should not be confused with outstanding gravimetric performance. An alloy does not need to exhibit record-high hydrogen content in order for the occupancy–transformation framework to remain useful. What matters is whether hydrogen can enter a host that is structurally coherent enough to sustain a stable sequence of uptake, structural response, and release. MgVCr therefore serves as a lower-bound success case: it is not an exceptional hydrogen-storage alloy, but it still behaves as a viable BCC-derived host rather than as a structurally incoherent absorber.

### 5.2. MgTiVCrFe: Pathway Breakdown Under Structural Incoherence

The contrasting case is MgTiVCrFe. Here, the alloy does not evolve into a comparably clear hydrogen-hosting state. Instead, the reported diffraction features are much broader and are accompanied by a stronger amorphous contribution, consistent with a mixed crystalline-amorphous host of much lower structural definition. Instead, after mechanical synthesis it exhibits coexistence of crystalline and amorphous regions, with a high level of amorphization and much weaker structural definition than MgVCr. This distinction proves decisive for hydrogen storage. At 350 °C, MgTiVCrFe absorbs only about 0.3 wt.% hydrogen and does so without a clearly defined pressure plateau; after cycling, the alloy shows partial decomposition from its initial structure, with possible formation of several secondary compounds [7]. In this case, the problem is not simply that the alloy performs worse than MgVCr. The more important point is that it does not sustain a coherent hydrogen-hosting route. The host state is too poorly defined and too unstable under hydrogen exposure for a robust occupancy hierarchy and a viable transformation pathway to persist. Its low uptake should therefore be interpreted not simply as a capacity deficit, but as the consequence of structural incoherence that interrupts the continuity between initial hydrogen accommodation and reversible release. By contrast, MgTiVCrFe does not maintain a similarly clean host ↔ hydride interconversion; its hydrogen uptake is better described as uptake accompanied by structural destabilization and partial decomposition.

**Figure 5:**
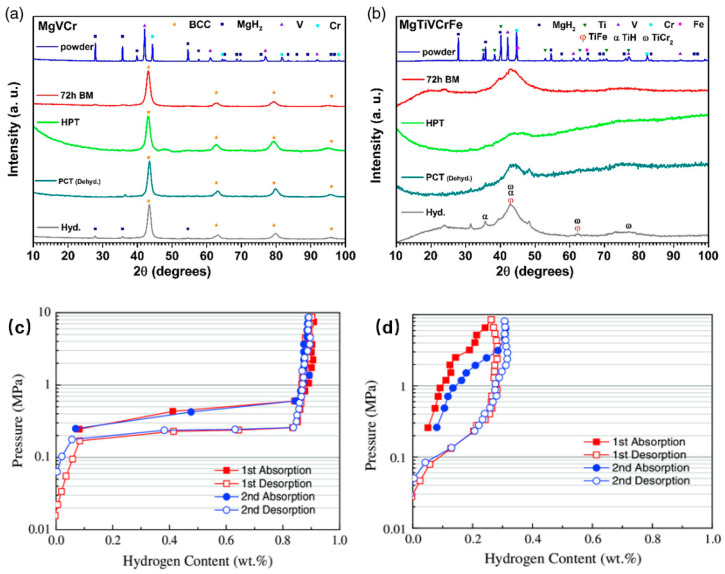
Comparison between a comparatively coherent and a disrupted hydrogen-hosting pathway in mechanically synthesized Mg-containing BCC-related alloys: (**a**) multi-state XRD patterns of MgVCr; (**b**) multi-state XRD patterns of MgTiVCrFe; (**c**) pressure-composition isotherm of MgVCr at 350 °C; and (**d**) pressure-composition isotherm of MgTiVCrFe at 350 °C. Adapted from Ref. [7].

This contrast is exactly what makes the pair so instructive. Both systems were designed and synthesized within a broadly similar BCC-related and mechanically activated framework, yet only one retains a pathway that remains structurally meaningful after hydrogen cycling. The difference therefore cannot be reduced to nominal chemistry alone. It reflects whether the alloy actually presents hydrogen with a crystallographically and chemically coherent host environment. Once that coherence is sufficiently degraded by amorphization, disorder, or instability, the BCC label loses most of its explanatory value. This contrast between a comparatively coherent pathway and a disrupted hydrogen-hosting route is summarized in Figure 5, clarifying why a nominally BCC-related design does not necessarily translate into a viable and reversible hydrogen-storage pathway [7].

### 5.3. Lightweighting, Nominal BCC Design, and Their Limits

The broader Mg-containing HEA literature reinforces the same conclusion. Lightweighting and BCC targeting are often treated as attractive design directions because they promise a better balance between gravimetric capacity and hydrogen-hosting framework. Yet the available reports show that the key difficulty is not simply to insert Mg or other light elements into a multicomponent alloy and retain a nominally BCC phase. The real challenge is whether the resulting alloy can preserve a pathway-compatible structural state once hydrogenation begins. This point has already been seen in hydrogen-induced phase-transition studies of MgZrTiFe_0.5_Co_0.5_Ni_0.5_, where the hydrogen-storage response is inseparable from a structural transformation of the host [12], and in subsequent work seeking to design single-BCC Mg-containing HEAs more systematically [17]. Related studies on Mg-containing multiprincipal-element alloys further show that gravimetric improvement and pathway integrity do not necessarily rise together [18,19]. In this sense, the design target is narrower than “make a lightweight BCC HEA.” What must be achieved is a structurally coherent BCC-derived host whose occupancy hierarchy and transformation route remain viable under real hydrogen exposure. This also explains why gravimetric improvement and pathway integrity do not necessarily rise together in Mg-containing multiprincipal-element alloys.

### 5.4. Pathway Integrity as a Practical Design Criterion

Taken together, these contrast cases sharpen the central claim of this perspective. A BCC-related alloy should not be judged only by nominal composition, by the presence of a BCC phase before hydrogenation, or even by initial hydrogen uptake alone. What matters more fundamentally is whether the alloy can support a coherent sequence linking initial site occupation to a stable and reversible hydrogenated structural state. MgVCr retains such a sequence in limited form, whereas MgTiVCrFe does not [7]. The broader Mg-containing HEA literature further suggests that this problem is not restricted to one isolated alloy pair, but reflects a more general design boundary for lightweight BCC-related systems [12,17,18,19]. The difference between viable and non-viable hydrogen hosts is therefore not only one of capacity or kinetics, but one of pathway integrity.

From a forward-looking design standpoint, pathway integrity in new materials is most likely to be anticipated not from nominal composition alone, but from a combination of structural and thermodynamic indicators. Candidate alloys should first retain a well-defined BCC-derived host after synthesis, rather than a heavily amorphized or weakly crystallized state. They should then exhibit a distributed but not destructive degree of local distortion, so that multiple hydrogen-accessible environments are created without collapsing overall lattice coherence. A further criterion is whether hydrogen uptake is likely to proceed through a limited number of energetically connected occupation states, rather than through broadly competing pathways that promote decomposition or structural dead ends. In practice, this means that future screening of BCC HEA hydrides should consider not only phase formation and storage capacity, but also crystallinity retention, local site hierarchy, and the likelihood of reversible phase switching under cycling conditions.

This conclusion also helps define the real design boundary of the class. The BCC label remains useful as a first structural filter, but it should not be mistaken for a sufficient design criterion. In practical terms, the usable design space of BCC HEA hydrides is narrower than the nominal compositional space from which they are drawn. What ultimately matters is whether the alloy realizes a hydrogen-hosting route that remains structurally coherent from early occupancy to reversible release. Once this criterion is accepted, failure cases become as informative as successful ones, because they reveal where the BCC occupancy–pathway logic begins to break down. That is why the next and final section must move beyond individual systems and toward a more explicit design outlook: if pathway integrity rather than phase label alone defines the useful class, then future progress must be framed as pathway engineering rather than composition screening.

## 6. Conclusions and Outlook

The central argument of this perspective is that hydrogen storage in BCC high-entropy alloys should not be interpreted primarily through nominal composition, nor even through average starting structure alone. What matters more fundamentally is how hydrogen is accommodated within the host lattice, how the hierarchy of accessible sites evolves as loading proceeds, and how that evolving occupancy redirects the alloy toward a specific hydrogenated structural route. Once the problem is framed in this way, the usual composition-centered description becomes insufficient. A BCC high-entropy alloy is not simply a multicomponent metallic host with broad compositional freedom. It is a structurally variable hydrogen-hosting framework in which interstitial topology, local strain, local chemistry, and hydrogen-induced phase switching are all coupled.

This shift in viewpoint changes the level at which BCC HEA hydrogen storage should be discussed. The decisive question is no longer only which elements are present, but where hydrogen first resides, which locally available sites remain favorable as hydrogen concentration increases, and whether the host can sustain a coherent structural route from early uptake to reversible release. In this sense, hydrogen occupancy and hydride-transformation pathway are not secondary details appended after capacity measurements. They are the missing middle layer between alloy design and hydrogen-storage response.

The systems considered in this article support that conclusion from different directions. Refractory alloys such as TiVZrNbHf and related systems show that high hydrogen uptake can emerge when the BCC host provides an occupancy landscape flexible enough to support mixed or evolving interstitial participation together with hydrogen-induced lattice transformation. Computational studies further indicate that occupancy is concentration-dependent and chemically nonuniform, rather than fixed by a single ideal interstitial model. By contrast, mechanically synthesized Mg-containing systems show that a nominally BCC-related compositional design does not guarantee an effective hydrogen-hosting pathway once amorphization, low crystallinity, or structural instability intervene. Taken together, these cases point to the same conclusion: what ultimately governs hydrogen-storage behavior is not the alloy label itself, but the integrity of the occupancy–transformation route that the alloy can support.

Future progress will depend less on expanding composition space indiscriminately and more on resolving the structural variables that govern storage. Priority should be given to occupancy-sensitive characterization, concentration-dependent modeling of local site evolution, and direct comparison of reversible and failure-prone transformation routes. In this framework, lower-performing alloys remain valuable because they help define where the BCC occupancy-pathway logic begins to break down. The broader goal is therefore not simply to identify more hydrogen-absorbing compositions, but to engineer hydrogen-hosting routes in which site accessibility, local chemistry, and phase switching remain coherent throughout cycling.

## Figures and Tables

**Table 1:** Representative BCC-related HEA systems linking local environment, lattice stress, site occupancy, and hydrogenation outcome.

System	Structural Feature/Local Environment	Stress or Site-Distribution Clue	Occupancy/Transformation Evidence	Hydrogen-Storage Implication	Ref.
TiVZrNbHf	Single-phase BCC refractory alloy with strong local lattice distortion and chemically diverse interstitial neighborhoods.	Broad local-environment distributions make tetrahedral and octahedral site energies non-equivalent.	Hydrogenation involves mixed site occupation and host-lattice transformation rather than simple rigid-lattice filling.	High capacity can emerge from a wide interstitial landscape, but reversibility depends on whether redistribution remains coordinated during cycling.	[3,11,12]
TiZrHfScMo/TiZrHfMoNb	Low-VEC, low-density BCC matrices with composition-dependent site openness.	Lower VEC can increase accessible interstitial space, but the local energy hierarchy remains composition sensitive.	Reported behavior includes both high uptake and hydride-phase segregation, showing that site availability alone does not guarantee reversible pathway coupling.	Useful for discussing the trade-off between capacity and structural stability in low-density HEAs.	[13,14]
TiVNbD5.7	Neutron-diffraction model system used to resolve hydrogen location directly.	Interatomic-distance analysis confirms that local geometry constrains which interstices remain favorable at high loading.	Predominantly tetrahedral occupation with residual octahedral involvement, depending on composition and hydrogen content.	Direct evidence that occupancy is conditional rather than fixed, and must be interpreted together with the evolving host lattice.	[15]
TiCrMnFeNi	High-pressure-synthesized BCC HEA used as a contrasting multicomponent host.	Configurational complexity alone is insufficient if the lattice does not maintain a coherent redistribution pathway under hydrogen insertion.	Reversible hydrogen uptake is achievable, but the storage response remains strongly pathway dependent.	Highlights that compositional complexity must be matched by structural compatibility.	[8]
MgVCr	Mg-containing BCC-related alloy with limited pathway coherence during hydrogenation and dehydrogenation.	Hydrogen insertion drives phase separation rather than well-synchronized redistribution within a stable multicomponent host.	Distinct hydride phases form on hydrogenation, and full restoration of the original host structure is not readily achieved on dehydrogenation.	Illustrates pathway failure: a nominally open parent lattice can still deliver poor reversibility when the reaction network fragments.	[7]
MgTiVCrFe	More compositionally complex Mg-containing alloy, but with stronger structural broadening and instability.	Additional elements modify the local environment, yet do not automatically preserve a coordinated absorption/desorption route.	Hydrogenation remains accompanied by structural destabilization, low reversible capacity, and poor cycling behavior.	Shows that increasing component count does not substitute for maintaining pathway integrity across the full reaction sequence.	[7]

## Data Availability

No new data were created or analyzed in this study. Data sharing is not applicable to this article.

## References

[1] Sakintuna B., Lamari-Darkrim F., Hirscher M. (2007). Metal hydride materials for solid hydrogen storage: A review. Int. J. Hydrogen Energy.

[2] Weidenthaler C., Felderhoff M. (2011). Solid-state hydrogen storage for mobile applications: Quo vadis?. Energy Environ. Sci..

[3] Marques F., Balcerzak M., Winkelmann F., Zepon G., Felderhoff M. (2021). Review and outlook on high-entropy alloys for hydrogen storage. Energy Environ. Sci..

[4] Kong L., Cheng B., Wan D., Xue Y. (2023). A review on BCC-structured high-entropy alloys for hydrogen storage. Front. Mater..

[5] Sahlberg M., Karlsson D., Zlotea C., Jansson U. (2016). Superior hydrogen storage in high entropy alloys. Sci. Rep..

[6] Hu J., Shen H., Jiang M., Gong H., Xiao H., Liu Z., Sun G., Zu X. (2019). A DFT study of hydrogen storage in high-entropy alloy TiZrHfScMo. Nanomaterials.

[7] de Marco M.O., Li Y., Li H.-W., Edalati K., Floriano R. (2020). Mechanical synthesis and hydrogen storage characterization of MgVCr and MgVTiCrFe high-entropy alloy. Adv. Eng. Mater..

[8] Nygård M.M., Ek G., Karlsson D., Sørby M.H., Sahlberg M., Hauback B.C. (2019). Counting electrons—A new approach to tailor the hydrogen sorption properties of high-entropy alloys. Acta Mater..

[9] Nygård M.M., Ek G., Karlsson D., Sahlberg M., Sørby M.H., Hauback B.C. (2019). Hydrogen storage in high-entropy alloys with varying degree of local lattice strain. Int. J. Hydrogen Energy.

[10] Hu J., Zhang J., Li M., Zhang S., Xiao H., Xie L., Sun G., Shen H., Zhou X., Li X. (2022). The origin of anomalous hydrogen occupation in high entropy alloys. J. Mater. Chem. A.

[11] Montero J., Ek G., Laversenne L., Nassif V., Sahlberg M., Zlotea C. (2021). How 10 at% Al addition in the Ti-V-Zr-Nb high-entropy alloy changes hydrogen sorption properties. Molecules.

[12] Zepon G., Leiva D.R., Strozi R.B., Bedoch A., Figueroa S.J.A., Ishikawa T.T., Botta W.J. (2018). Hydrogen-induced phase transition of MgZrTiFe0.5Co0.5Ni0.5 high entropy alloy. Int. J. Hydrogen Energy.

[13] Ek G., Nygård M.M., Pavan A.F., Montero J., Henry P.F., Sørby M.H., Sahlberg M., Zlotea C. (2021). Elucidating the effects of the composition on hydrogen sorption in TiVZrNbHf-based high-entropy alloys. Inorg. Chem..

[14] Nygård M.M., Sławiński W.A., Ek G., Sørby M.H., Sahlberg M., Keen D.A., Hauback B.C. (2020). Local order in high-entropy alloys and associated deuterides—A total scattering and Reverse Monte Carlo study. Acta Mater..

[15] Hu J., Zhang J., Xiao H., Xie L., Sun G., Shen H., Zhou X., Li X., Li P., Vitos L. (2020). A density functional theory study of the hydrogen absorption in high entropy alloy TiZrHfMoNb. Inorg. Chem..

[16] Muñiz C.M., Hussain T., Liu W., Aguey-Zinsou K.-F. (2024). On the interplay of tetrahedral and octahedral hydrogen absorption sites in the Ti28V20Cr52 alloy: First principles study. Comput. Condens. Matter.

[17] Strozi R.B., Leiva D.R., Huot J., Botta W.J., Zepon G. (2021). An approach to design single BCC Mg-containing high entropy alloys for hydrogen storage applications. Int. J. Hydrogen Energy.

[18] Marques F., Pinto H.C., Figueroa S.J.A., Winkelmann F., Felderhoff M., Botta W.J., Zepon G. (2020). Mg-containing multi-principal element alloys for hydrogen storage: A study of the MgTiNbCr_0.5_Mn_0.5_Ni_0.5_ and Mg_0.68_TiNbNi_0.55_ compositions. Int. J. Hydrogen Energy.

[19] Montero J., Ek G., Sahlberg M., Zlotea C. (2021). Improving the hydrogen cycling properties by Mg addition in Ti-V-Zr-Nb refractory high entropy alloy. Scr. Mater..

